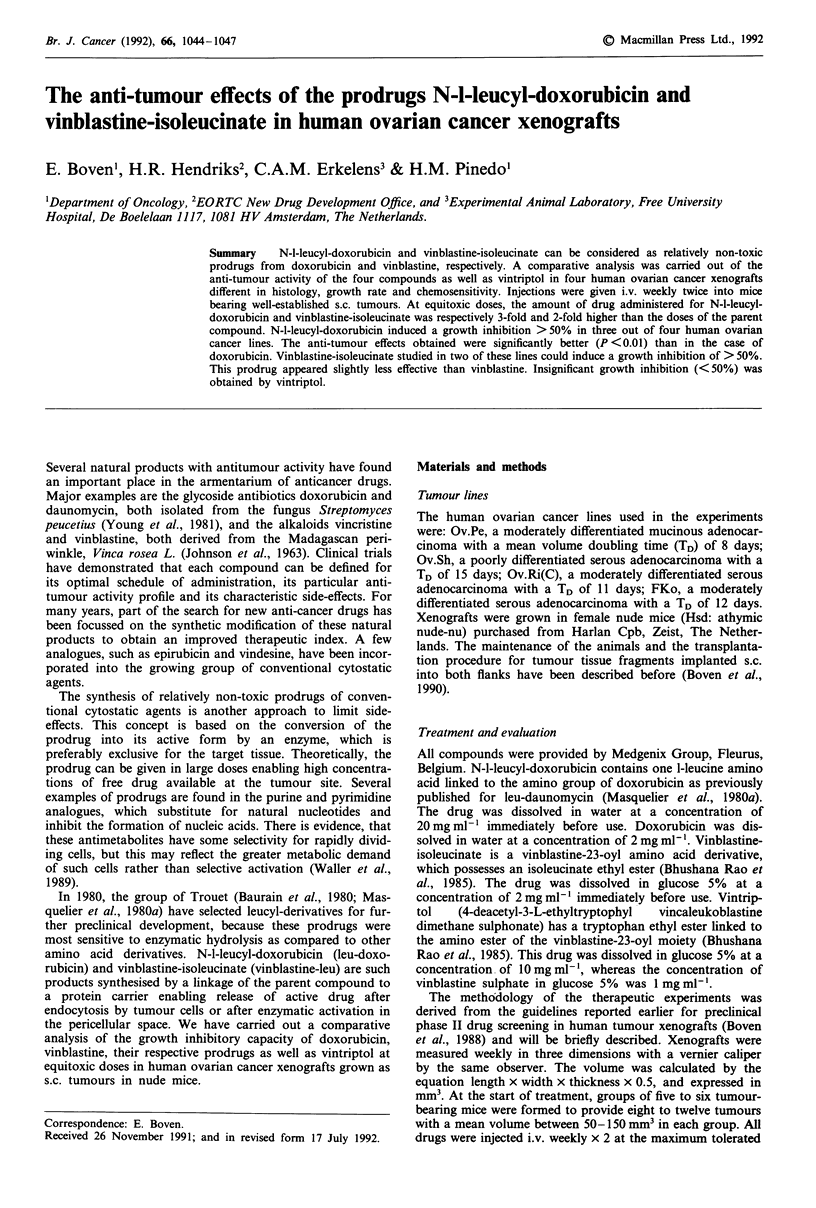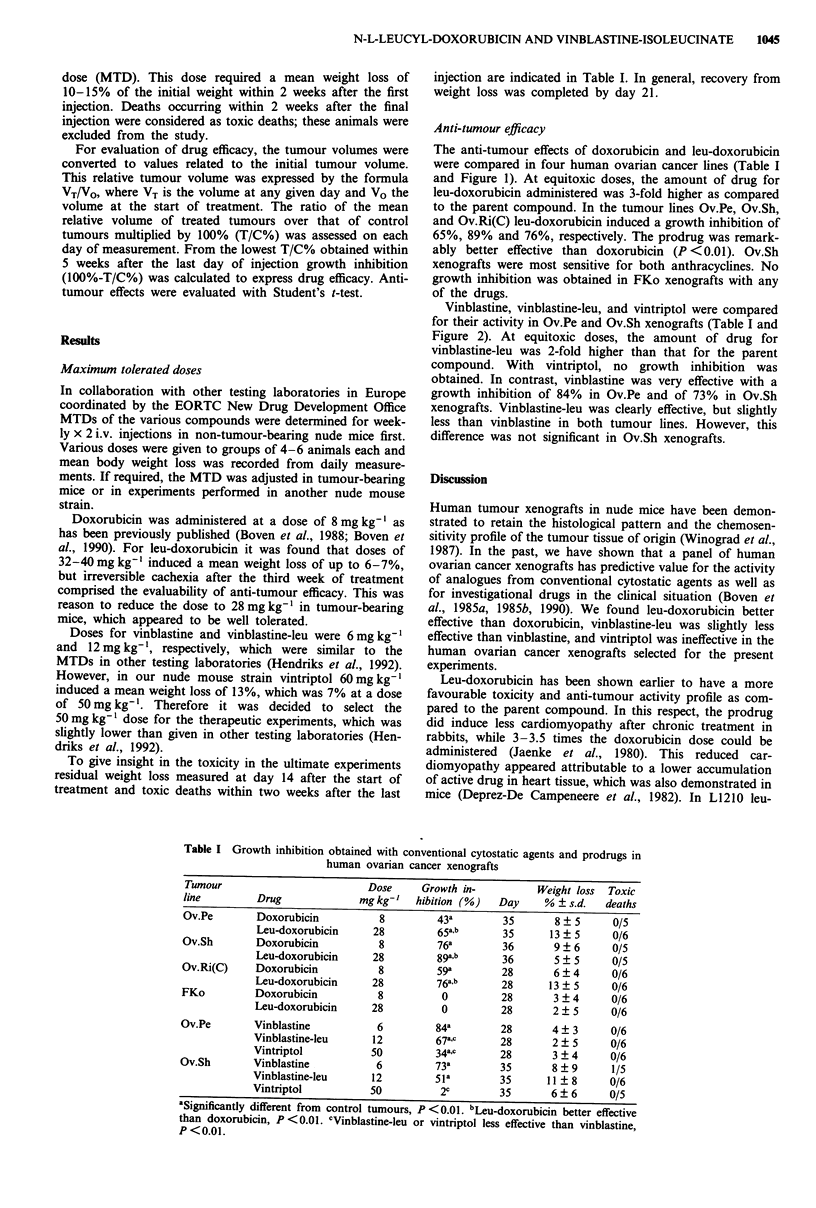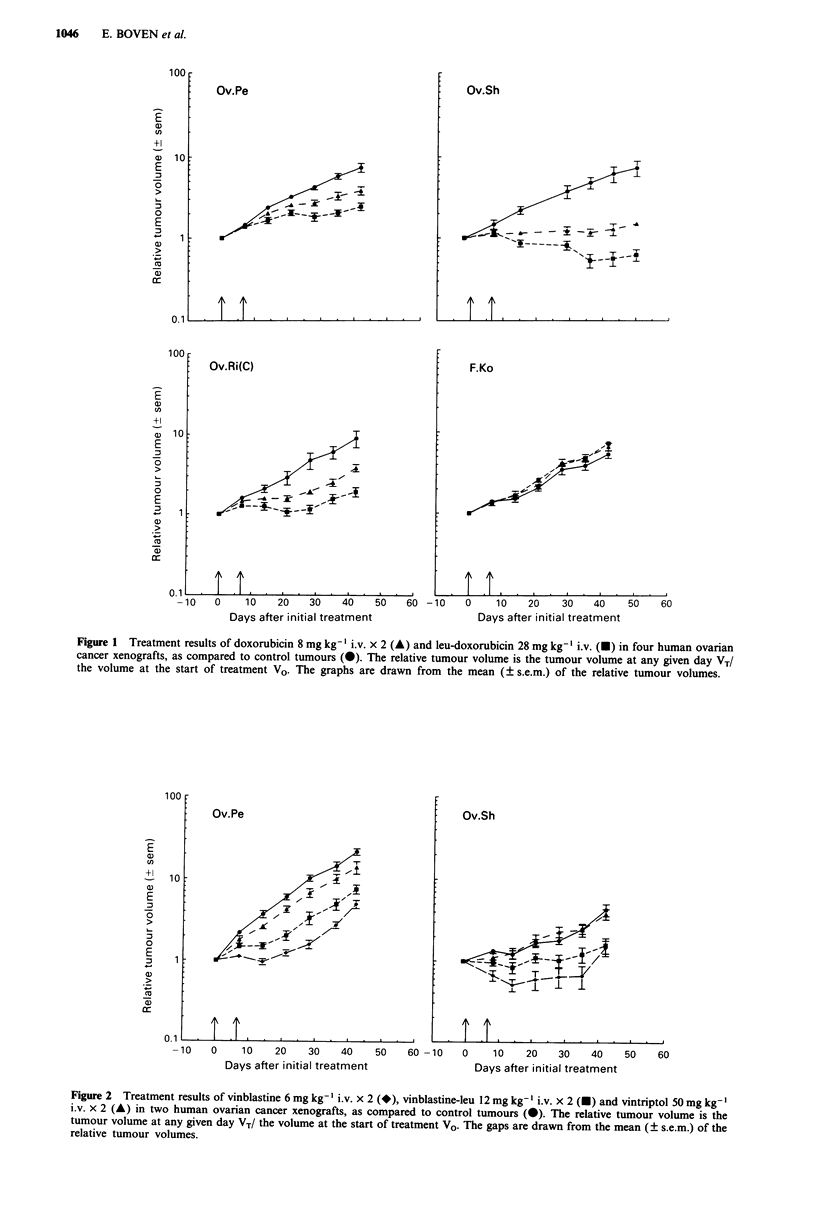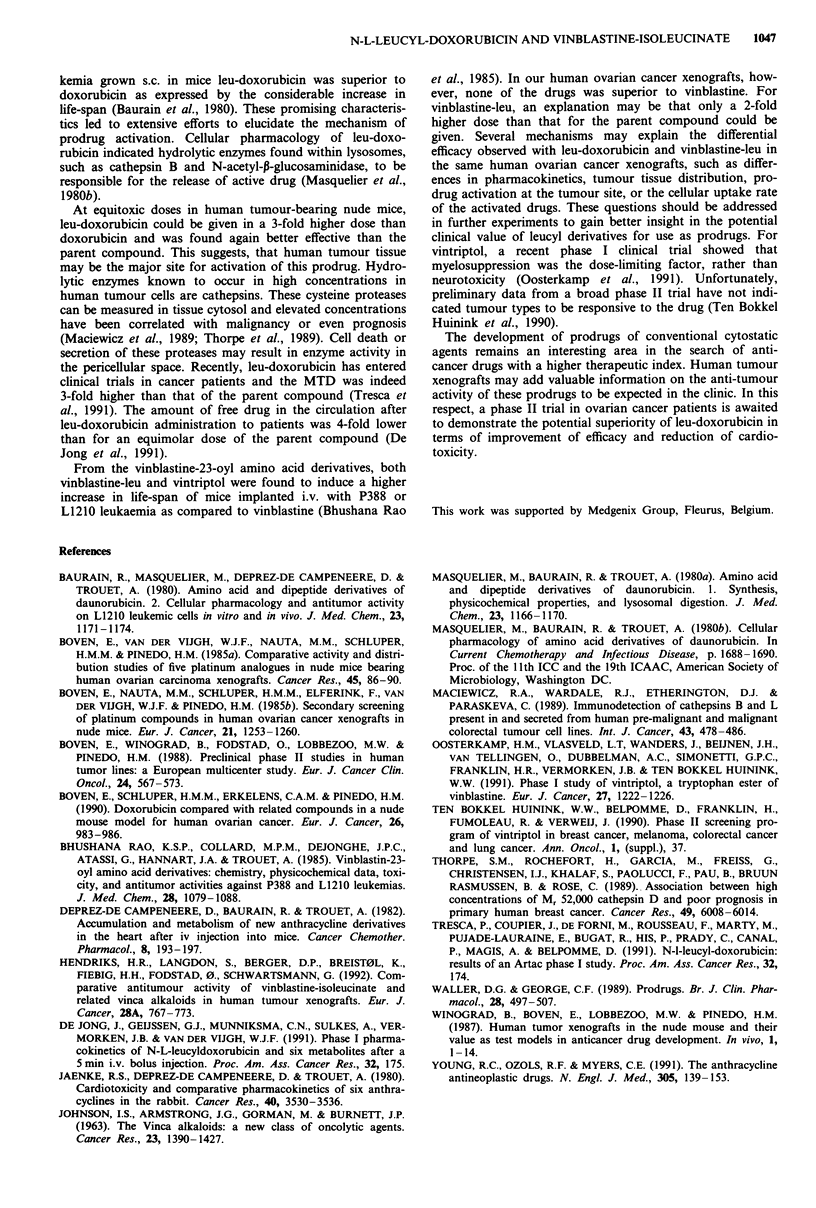# The anti-tumour effects of the prodrugs N-l-leucyl-doxorubicin and vinblastine-isoleucinate in human ovarian cancer xenografts.

**DOI:** 10.1038/bjc.1992.407

**Published:** 1992-12

**Authors:** E. Boven, H. R. Hendriks, C. A. Erkelens, H. M. Pinedo

**Affiliations:** Department of Oncology, Free University Hospital, Amsterdam, The Netherlands.

## Abstract

N-l-leucyl-doxorubicin and vinblastine-isoleucinate can be considered as relatively non-toxic prodrugs from doxorubicin and vinblastine, respectively. A comparative analysis was carried out of the anti-tumour activity of the four compounds as well as vintriptol in four human ovarian cancer xenografts different in histology, growth rate and chemosensitivity. Injections were given i.v. weekly twice into mice bearing well-established s.c. tumours. At equitoxic doses, the amount of drug administered for N-l-leucyl-doxorubicin and vinblastine-isoleucinate was respectively 3-fold and 2-fold higher than the doses of the parent compound. N-l-leucyl-doxorubicin induced a growth inhibition > 50% in three out of four human ovarian cancer lines. The anti-tumour effects obtained were significantly better (P < 0.01) than in the case of doxorubicin. Vinblastine-isoleucinate studied in two of these lines could induce a growth inhibition of > 50%. This prodrug appeared slightly less effective than vinblastine. Insignificant growth inhibition (< 50%) was obtained by vintriptol.


Br. J. Cancer (1992), 66, 1044-1047 © Macmillan Press Ltd., 1992

### The anti-tumour effects of the prodrugs N-l-leucyl-doxorubicin and vinblastine-isoleucinate in human ovarian cancer xenografts

E. Boven[1], H.R. Hendriks[2], C.A.M. Erkelens[3] & H.M. Pinedo[1]

[1]*Department of Oncology,* [2]*EORTC New Drug Development Office, and* [3]*Experimental Animal Laboratory, Free University Hospital, De Boelelaan 1117, 1081 HV Amsterdam, The Netherlands.*

**Summary** N-l-leucyl-doxorubicin and vinblastine-isoleucinate can be considered as relatively non-toxic prodrugs from doxorubicin and vinblastine, respectively. A comparative analysis was carried out of the anti-tumour activity of the four compounds as well as vintriptol in four human ovarian cancer xenografts different in histology, growth rate and chemosensitivity. Injections were given i.v. weekly twice into mice bearing well-established s.c. tumours. At equitoxic doses, the amount of drug administered for N-l-leucyl-doxorubicin and vinblastine-isoleucinate was respectively 3-fold and 2-fold higher than the doses of the parent compound. N-l-leucyl-doxorubicin induced a growth inhibition $>50\%$ in three out of four human ovarian cancer lines. The anti-tumour effects obtained were significantly better ($P<0.01$) than in the case of doxorubicin. Vinblastine-isoleucinate studied in two of these lines could induce a growth inhibition of $>50\%$. This prodrug appeared slightly less effective than vinblastine. Insignificant growth inhibition ($<50\%$) was obtained by vintriptol.

Several natural products with antitumour activity have found an important place in the armentarium of anticancer drugs. Major examples are the glycoside antibiotics doxorubicin and daunomycin, both isolated from the fungus *Streptomyces peucetius* (Young *et al.*, 1981), and the alkaloids vincristine and vinblastine, both derived from the Madagascan periwinkle, *Vinca rosea L.* (Johnson *et al.*, 1963). Clinical trials have demonstrated that each compound can be defined for its optimal schedule of administration, its particular anti-tumour activity profile and its characteristic side-effects. For many years, part of the search for new anti-cancer drugs has been focussed on the synthetic modification of these natural products to obtain an improved therapeutic index. A few analogues, such as epirubicin and vindesine, have been incorporated into the growing group of conventional cytostatic agents.

The synthesis of relatively non-toxic prodrugs of conventional cytostatic agents is another approach to limit side-effects. This concept is based on the conversion of the prodrug into its active form by an enzyme, which is preferably exclusive for the target tissue. Theoretically, the prodrug can be given in large doses enabling high concentrations of free drug available at the tumour site. Several examples of prodrugs are found in the purine and pyrimidine analogues, which substitute for natural nucleotides and inhibit the formation of nucleic acids. There is evidence, that these antimetabolites have some selectivity for rapidly dividing cells, but this may reflect the greater metabolic demand of such cells rather than selective activation (Waller *et al.*, 1989).

In 1980, the group of Trouet (Baurain *et al.*, 1980; Masquelier *et al.*, 1980*a*) have selected leucyl-derivatives for further preclinical development, because these prodrugs were most sensitive to enzymatic hydrolysis as compared to other amino acid derivatives. N-l-leucyl-doxorubicin (leu-doxorubicin) and vinblastine-isoleucinate (vinblastine-leu) are such products synthesised by a linkage of the parent compound to a protein carrier enabling release of active drug after endocytosis by tumour cells or after enzymatic activation in the pericellular space. We have carried out a comparative analysis of the growth inhibitory capacity of doxorubicin, vinblastine, their respective prodrugs as well as vintriptol at equitoxic doses in human ovarian cancer xenografts grown as s.c. tumours in nude mice.

Correspondence: E. Boven.
Received 26 November 1991; and in revised form 17 July 1992.

### Materials and methods

### *Tumour lines*

The human ovarian cancer lines used in the experiments were: Ov.Pe, a moderately differentiated mucinous adenocarcinoma with a mean volume doubling time ($T_D$) of 8 days; Ov.Sh, a poorly differentiated serous adenocarcinoma with a $T_D$ of 15 days; Ov.Ri(C), a moderately differentiated serous adenocarcinoma with a $T_D$ of 11 days; FKo, a moderately differentiated serous adenocarcinoma with a $T_D$ of 12 days. Xenografts were grown in female nude mice (Hsd: athymic nude-nu) purchased from Harlan Cpb, Zeist, The Netherlands. The maintenance of the animals and the transplantation procedure for tumour tissue fragments implanted s.c. into both flanks have been described before (Boven *et al.*, 1990).

### *Treatment and evaluation*

All compounds were provided by Medgenix Group, Fleurus, Belgium. N-l-leucyl-doxorubicin contains one l-leucine amino acid linked to the amino group of doxorubicin as previously published for leu-daunomycin (Masquelier *et al.*, 1980*a*). The drug was dissolved in water at a concentration of 20 mg ml$^{-1}$ immediately before use. Doxorubicin was dissolved in water at a concentration of 2 mg ml$^{-1}$. Vinblastine-isoleucinate is a vinblastine-23-oyl amino acid derivative, which possesses an isoleucinate ethyl ester (Bhushana Rao *et al.*, 1985). The drug was dissolved in glucose 5% at a concentration of 2 mg ml$^{-1}$ immediately before use. Vintriptol (4-deacetyl-3-L-ethyltryptophyl vincaleukoblastine dimethane sulphonate) has a tryptophan ethyl ester linked to the amino ester of the vinblastine-23-oyl moiety (Bhushana Rao *et al.*, 1985). This drug was dissolved in glucose 5% at a concentration of 10 mg ml$^{-1}$, whereas the concentration of vinblastine sulphate in glucose 5% was 1 mg ml$^{-1}$.

The methodology of the therapeutic experiments was derived from the guidelines reported earlier for preclinical phase II drug screening in human tumour xenografts (Boven *et al.*, 1988) and will be briefly described. Xenografts were measured weekly in three dimensions with a vernier caliper by the same observer. The volume was calculated by the equation length × width × thickness × 0.5, and expressed in mm$^3$. At the start of treatment, groups of five to six tumour-bearing mice were formed to provide eight to twelve tumours with a mean volume between 50–150 mm$^3$ in each group. All drugs were injected i.v. weekly × 2 at the maximum tolerated dose (MTD). This dose required a mean weight loss of 10–15% of the initial weight within 2 weeks after the first injection. Deaths occurring within 2 weeks after the final injection were considered as toxic deaths; these animals were excluded from the study.

For evaluation of drug efficacy, the tumour volumes were converted to values related to the initial tumour volume. This relative tumour volume was expressed by the formula $V_T/V_O$, where $V_T$ is the volume at any given day and $V_O$ the volume at the start of treatment. The ratio of the mean relative volume of treated tumours over that of control tumours multiplied by 100% (T/C%) was assessed on each day of measurement. From the lowest T/C% obtained within 5 weeks after the last day of injection growth inhibition (100%-T/C%) was calculated to express drug efficacy. Anti-tumour effects were evaluated with Student's *t*-test.

### Results

### *Maximum tolerated doses*

In collaboration with other testing laboratories in Europe coordinated by the EORTC New Drug Development Office MTDs of the various compounds were determined for weekly × 2 i.v. injections in non-tumour-bearing nude mice first. Various doses were given to groups of 4–6 animals each and mean body weight loss was recorded from daily measurements. If required, the MTD was adjusted in tumour-bearing mice or in experiments performed in another nude mouse strain.

Doxorubicin was administered at a dose of $8 \text{ mg kg}^{-1}$ as has been previously published (Boven *et al.*, 1988; Boven *et al.*, 1990). For leu-doxorubicin it was found that doses of $32-40 \text{ mg kg}^{-1}$ induced a mean weight loss of up to 6–7%, but irreversible cachexia after the third week of treatment comprised the evaluability of anti-tumour efficacy. This was reason to reduce the dose to $28 \text{ mg kg}^{-1}$ in tumour-bearing mice, which appeared to be well tolerated.

Doses for vinblastine and vinblastine-leu were $6 \text{ mg kg}^{-1}$ and $12 \text{ mg kg}^{-1}$, respectively, which were similar to the MTDs in other testing laboratories (Hendriks *et al.*, 1992). However, in our nude mouse strain vintriptol $60 \text{ mg kg}^{-1}$ induced a mean weight loss of 13%, which was 7% at a dose of $50 \text{ mg kg}^{-1}$. Therefore it was decided to select the $50 \text{ mg kg}^{-1}$ dose for the therapeutic experiments, which was slightly lower than given in other testing laboratories (Hendriks *et al.*, 1992).

To give insight in the toxicity in the ultimate experiments residual weight loss measured at day 14 after the start of treatment and toxic deaths within two weeks after the last injection are indicated in Table I. In general, recovery from weight loss was completed by day 21.

### *Anti-tumour efficacy*

The anti-tumour effects of doxorubicin and leu-doxorubicin were compared in four human ovarian cancer lines (Table I and Figure 1). At equitoxic doses, the amount of drug for leu-doxorubicin administered was 3-fold higher as compared to the parent compound. In the tumour lines Ov.Pe, Ov.Sh, and Ov.Ri(C) leu-doxorubicin induced a growth inhibition of 65%, 89% and 76%, respectively. The prodrug was remarkably better effective than doxorubicin ($P < 0.01$). Ov.Sh xenografts were most sensitive for both anthracyclines. No growth inhibition was obtained in FKo xenografts with any of the drugs.

Vinblastine, vinblastine-leu, and vintriptol were compared for their activity in Ov.Pe and Ov.Sh xenografts (Table I and Figure 2). At equitoxic doses, the amount of drug for vinblastine-leu was 2-fold higher than that for the parent compound. With vintriptol, no growth inhibition was obtained. In contrast, vinblastine was very effective with a growth inhibition of 84% in Ov.Pe and of 73% in Ov.Sh xenografts. Vinblastine-leu was clearly effective, but slightly less than vinblastine in both tumour lines. However, this difference was not significant in Ov.Sh xenografts.

### Discussion

Human tumour xenografts in nude mice have been demonstrated to retain the histological pattern and the chemosensitivity profile of the tumour tissue of origin (Winograd *et al.*, 1987). In the past, we have shown that a panel of human ovarian cancer xenografts has predictive value for the activity of analogues from conventional cytostatic agents as well as for investigational drugs in the clinical situation (Boven *et al.*, 1985*a*, 1985*b*, 1990). We found leu-doxorubicin better effective than doxorubicin, vinblastine-leu was slightly less effective than vinblastine, and vintriptol was ineffective in the human ovarian cancer xenografts selected for the present experiments.

Leu-doxorubicin has been shown earlier to have a more favourable toxicity and anti-tumour activity profile as compared to the parent compound. In this respect, the prodrug did induce less cardiomyopathy after chronic treatment in rabbits, while 3–3.5 times the doxorubicin dose could be administered (Jaenke *et al.*, 1980). This reduced cardiomyopathy appeared attributable to a lower accumulation of active drug in heart tissue, which was also demonstrated in mice (Deprez-De Campeneere *et al.*, 1982). In L1210 leu-

**Table I** Growth inhibition obtained with conventional cytostatic agents and prodrugs in human ovarian cancer xenografts

| *Tumour line* | *Drug* | *Dose* $mg\ kg^{-1}$ | *Growth inhibition (%)* | *Day* | *Weight loss % ± s.d.* | *Toxic deaths* |
|---|---|---|---|---|---|---|
| Ov.Pe | Doxorubicin | 8 | 43[a] | 35 | 8 ± 5 | 0/5 |
| | Leu-doxorubicin | 28 | 65[a,b] | 35 | 13 ± 5 | 0/6 |
| Ov.Sh | Doxorubicin | 8 | 76[a] | 36 | 9 ± 6 | 0/5 |
| | Leu-doxorubicin | 28 | 89[a,b] | 36 | 5 ± 5 | 0/5 |
| Ov.Ri(C) | Doxorubicin | 8 | 59[a] | 28 | 6 ± 4 | 0/6 |
| | Leu-doxorubicin | 28 | 76[a,b] | 28 | 13 ± 5 | 0/6 |
| FKo | Doxorubicin | 8 | 0 | 28 | 3 ± 4 | 0/6 |
| | Leu-doxorubicin | 28 | 0 | 28 | 2 ± 5 | 0/6 |
| Ov.Pe | Vinblastine | 6 | 84[a] | 28 | 4 ± 3 | 0/6 |
| | Vinblastine-leu | 12 | 67[a,c] | 28 | 2 ± 5 | 0/6 |
| | Vintriptol | 50 | 34[a,c] | 28 | 3 ± 4 | 0/6 |
| Ov.Sh | Vinblastine | 6 | 73[a] | 35 | 8 ± 9 | 1/5 |
| | Vinblastine-leu | 12 | 51[a] | 35 | 11 ± 8 | 0/6 |
| | Vintriptol | 50 | 2[c] | 35 | 6 ± 6 | 0/5 |

[a]Significantly different from control tumours, $P < 0.01$. [b]Leu-doxorubicin better effective than doxorubicin, $P < 0.01$. [c]Vinblastine-leu or vintriptol less effective than vinblastine, $P < 0.01$.

**Figure 1** Treatment results of doxorubicin 8 mg $kg^{-1}$ i.v. × 2 (▲) and leu-doxorubicin 28 mg $kg^{-1}$ i.v. (■) in four human ovarian cancer xenografts, as compared to control tumours (●). The relative tumour volume is the tumour volume at any given day $V_T$/ the volume at the start of treatment $V_O$. The graphs are drawn from the mean (± s.e.m.) of the relative tumour volumes.

**Figure 2** Treatment results of vinblastine 6 mg $kg^{-1}$ i.v. × 2 (◆), vinblastine-leu 12 mg $kg^{-1}$ i.v. × 2 (■) and vintriptol 50 mg $kg^{-1}$ i.v. × 2 (▲) in two human ovarian cancer xenografts, as compared to control tumours (●). The relative tumour volume is the tumour volume at any given day $V_T$/ the volume at the start of treatment $V_O$. The gaps are drawn from the mean (± s.e.m.) of the relative tumour volumes.

kemia grown s.c. in mice leu-doxorubicin was superior to doxorubicin as expressed by the considerable increase in life-span (Baurain *et al.*, 1980). These promising characteristics led to extensive efforts to elucidate the mechanism of prodrug activation. Cellular pharmacology of leu-doxorubicin indicated hydrolytic enzymes found within lysosomes, such as cathepsin B and N-acetyl-β-glucosaminidase, to be responsible for the release of active drug (Masquelier *et al.*, 1980*b*).

At equitoxic doses in human tumour-bearing nude mice, leu-doxorubicin could be given in a 3-fold higher dose than doxorubicin and was found again better effective than the parent compound. This suggests, that human tumour tissue may be the major site for activation of this prodrug. Hydrolytic enzymes known to occur in high concentrations in human tumour cells are cathepsins. These cysteine proteases can be measured in tissue cytosol and elevated concentrations have been correlated with malignancy or even prognosis (Maciewicz *et al.*, 1989; Thorpe *et al.*, 1989). Cell death or secretion of these proteases may result in enzyme activity in the pericellular space. Recently, leu-doxorubicin has entered clinical trials in cancer patients and the MTD was indeed 3-fold higher than that of the parent compound (Tresca *et al.*, 1991). The amount of free drug in the circulation after leu-doxorubicin administration to patients was 4-fold lower than for an equimolar dose of the parent compound (De Jong *et al.*, 1991).

From the vinblastine-23-oyl amino acid derivatives, both vinblastine-leu and vintriptol were found to induce a higher increase in life-span of mice implanted i.v. with P388 or L1210 leukaemia as compared to vinblastine (Bhushana Rao *et al.*, 1985). In our human ovarian cancer xenografts, however, none of the drugs was superior to vinblastine. For vinblastine-leu, an explanation may be that only a 2-fold higher dose than that for the parent compound could be given. Several mechanisms may explain the differential efficacy observed with leu-doxorubicin and vinblastine-leu in the same human ovarian cancer xenografts, such as differences in pharmacokinetics, tumour tissue distribution, prodrug activation at the tumour site, or the cellular uptake rate of the activated drugs. These questions should be addressed in further experiments to gain better insight in the potential clinical value of leucyl derivatives for use as prodrugs. For vintriptol, a recent phase I clinical trial showed that myelosuppression was the dose-limiting factor, rather than neurotoxicity (Oosterkamp *et al.*, 1991). Unfortunately, preliminary data from a broad phase II trial have not indicated tumour types to be responsive to the drug (Ten Bokkel Huinink *et al.*, 1990).

The development of prodrugs of conventional cytostatic agents remains an interesting area in the search of anticancer drugs with a higher therapeutic index. Human tumour xenografts may add valuable information on the anti-tumour activity of these prodrugs to be expected in the clinic. In this respect, a phase II trial in ovarian cancer patients is awaited to demonstrate the potential superiority of leu-doxorubicin in terms of improvement of efficacy and reduction of cardiotoxicity.

This work was supported by Medgenix Group, Fleurus, Belgium.

### References

BAURAIN, R., MASQUELIER, M., DEPREZ-DE CAMPENEERE, D. & TROUET, A. (1980). Amino acid and dipeptide derivatives of daunorubicin. 2. Cellular pharmacology and antitumor activity on L1210 leukemic cells *in vitro* and *in vivo*. *J. Med. Chem.*, **23**, 1171–1174.

BOVEN, E., VAN DER VIJGH, W.J.F., NAUTA, M.M., SCHLUPER, H.M.M. & PINEDO, H.M. (1985*a*). Comparative activity and distribution studies of five platinum analogues in nude mice bearing human ovarian carcinoma xenografts. *Cancer Res.*, **45**, 86–90.

BOVEN, E., NAUTA, M.M., SCHLUPER, H.M.M., ELFERINK, F., VAN DER VIJGH, W.J.F. & PINEDO, H.M. (1985*b*). Secondary screening of platinum compounds in human ovarian cancer xenografts in nude mice. *Eur. J. Cancer*, **21**, 1253–1260.

BOVEN, E., WINOGRAD, B., FODSTAD, O., LOBBEZOO, M.W. & PINEDO, H.M. (1988). Preclinical phase II studies in human tumor lines: a European multicenter study. *Eur. J. Cancer Clin. Oncol.*, **24**, 567–573.

BOVEN, E., SCHLUPER, H.M.M., ERKELENS, C.A.M. & PINEDO, H.M. (1990). Doxorubicin compared with related compounds in a nude mouse model for human ovarian cancer. *Eur. J. Cancer*, **26**, 983–986.

BHUSHANA RAO, K.S.P., COLLARD, M.P.M., DEJONGHE, J.P.C., ATASSI, G., HANNART, J.A. & TROUET, A. (1985). Vinblastin-23-oyl amino acid derivatives: chemistry, physicochemical data, toxicity, and antitumor activities against P388 and L1210 leukemias. *J. Med. Chem.*, **28**, 1079–1088.

DEPREZ-DE CAMPENEERE, D., BAURAIN, R. & TROUET, A. (1982). Accumulation and metabolism of new anthracycline derivatives in the heart after iv injection into mice. *Cancer Chemother. Pharmacol.*, **8**, 193–197.

HENDRIKS, H.R., LANGDON, S., BERGER, D.P., BREISTØL, K., FIEBIG, H.H., FODSTAD, Ø., SCHWARTSMANN, G. (1992). Comparative antitumour activity of vinblastine-isoleucinate and related vinca alkaloids in human tumour xenografts. *Eur. J. Cancer*, **28A**, 767–773.

DE JONG, J., GEIJSSEN, G.J., MUNNIKSMA, C.N., SULKES, A., VERMORKEN, J.B. & VAN DER VIJGH, W.J.F. (1991). Phase I pharmacokinetics of N-L-leucyldoxorubicin and six metabolites after a 5 min i.v. bolus injection. *Proc. Am. Ass. Cancer Res.*, **32**, 175.

JAENKE, R.S., DEPREZ-DE CAMPENEERE, D. & TROUET, A. (1980). Cardiotoxicity and comparative pharmacokinetics of six anthracyclines in the rabbit. *Cancer Res.*, **40**, 3530–3536.

JOHNSON, I.S., ARMSTRONG, J.G., GORMAN, M. & BURNETT, J.P. (1963). The Vinca alkaloids: a new class of oncolytic agents. *Cancer Res.*, **23**, 1390–1427.

MASQUELIER, M., BAURAIN, R. & TROUET, A. (1980*a*). Amino acid and dipeptide derivatives of daunorubicin. 1. Synthesis, physicochemical properties, and lysosomal digestion. *J. Med. Chem.*, **23**, 1166–1170.

MASQUELIER, M., BAURAIN, R. & TROUET, A. (1980*b*). Cellular pharmacology of amino acid derivatives of daunorubicin. In *Current Chemotherapy and Infectious Disease*, p. 1688–1690. Proc. of the 11th ICC and the 19th ICAAC, American Society of Microbiology, Washington DC.

MACIEWICZ, R.A., WARDALE, R.J., ETHERINGTON, D.J. & PARASKEVA, C. (1989). Immunodetection of cathepsins B and L present in and secreted from human pre-malignant and malignant colorectal tumour cell lines. *Int. J. Cancer*, **43**, 478–486.

OOSTERKAMP, H.M., VLASVELD, L.T, WANDERS, J., BEIJNEN, J.H., VAN TELLINGEN, O., DUBBELMAN, A.C., SIMONETTI, G.P.C., FRANKLIN, H.R., VERMORKEN, J.B. & TEN BOKKEL HUININK, W.W. (1991). Phase I study of vintriptol, a tryptophan ester of vinblastine. *Eur. J. Cancer*, **27**, 1222–1226.

TEN BOKKEL HUININK, W.W., BELPOMME, D., FRANKLIN, H., FUMOLEAU, R. & VERWEIJ, J. (1990). Phase II screening program of vintriptol in breast cancer, melanoma, colorectal cancer and lung cancer. *Ann. Oncol.*, **1**, (suppl.), 37.

THORPE, S.M., ROCHEFORT, H., GARCIA, M., FREISS, G., CHRISTENSEN, I.J., KHALAF, S., PAOLUCCI, F., PAU, B., BRUUN RASMUSSEN, B. & ROSE, C. (1989).. Association between high concentrations of $M_r$ 52,000 cathepsin D and poor prognosis in primary human breast cancer. *Cancer Res.*, **49**, 6008–6014.

TRESCA, P., COUPIER, J., DE FORNI, M., ROUSSEAU, F., MARTY, M., PUJADE-LAURAINE, E., BUGAT, R., HIS, P., PRADY, C., CANAL, P., MAGIS, A. & BELPOMME, D. (1991). N-l-leucyl-doxorubicin: results of an Artac phase I study. *Proc. Am. Ass. Cancer Res.*, **32**, 174.

WALLER, D.G. & GEORGE, C.F. (1989). Prodrugs. *Br. J. Clin. Pharmacol.*, **28**, 497–507.

WINOGRAD, B., BOVEN, E., LOBBEZOO, M.W. & PINEDO, H.M. (1987). Human tumor xenografts in the nude mouse and their value as test models in anticancer drug development. *In vivo*, **1**, 1–14.

YOUNG, R.C., OZOLS, R.F. & MYERS, C.E. (1991). The anthracycline antineoplastic drugs. *N. Engl. J. Med.*, **305**, 139–153.